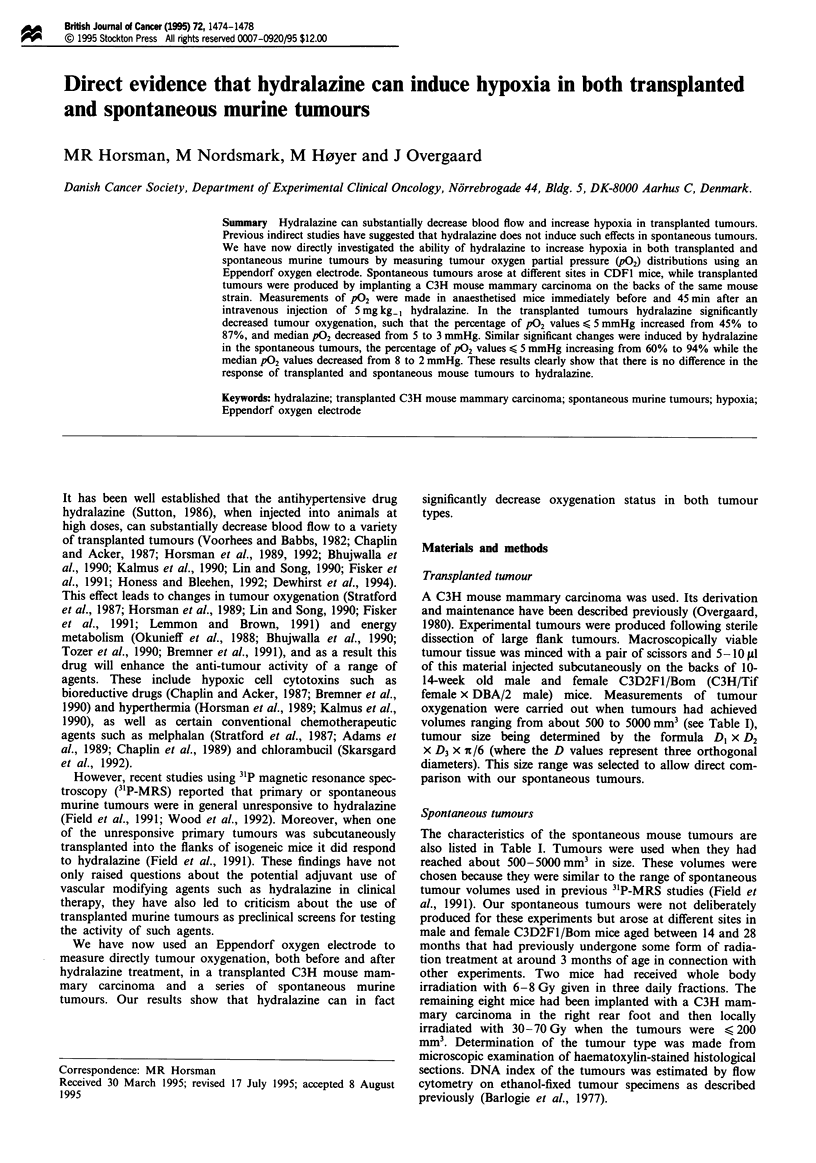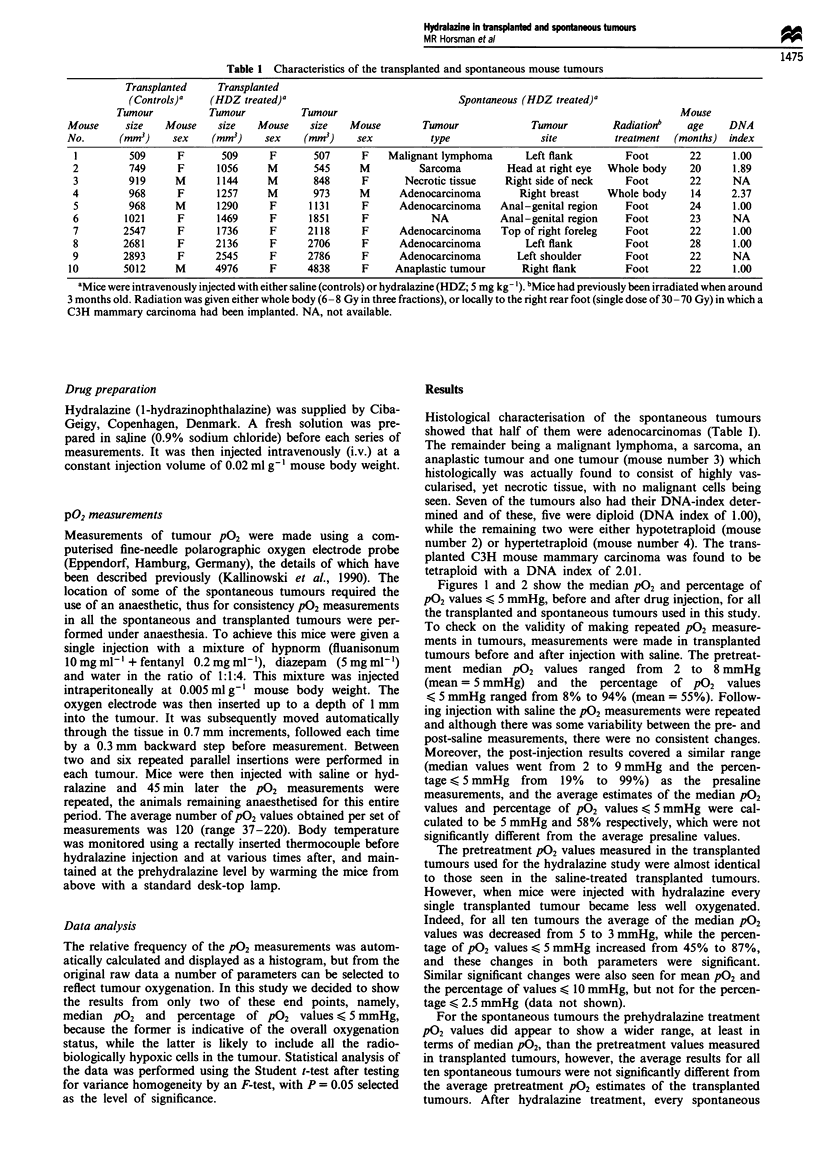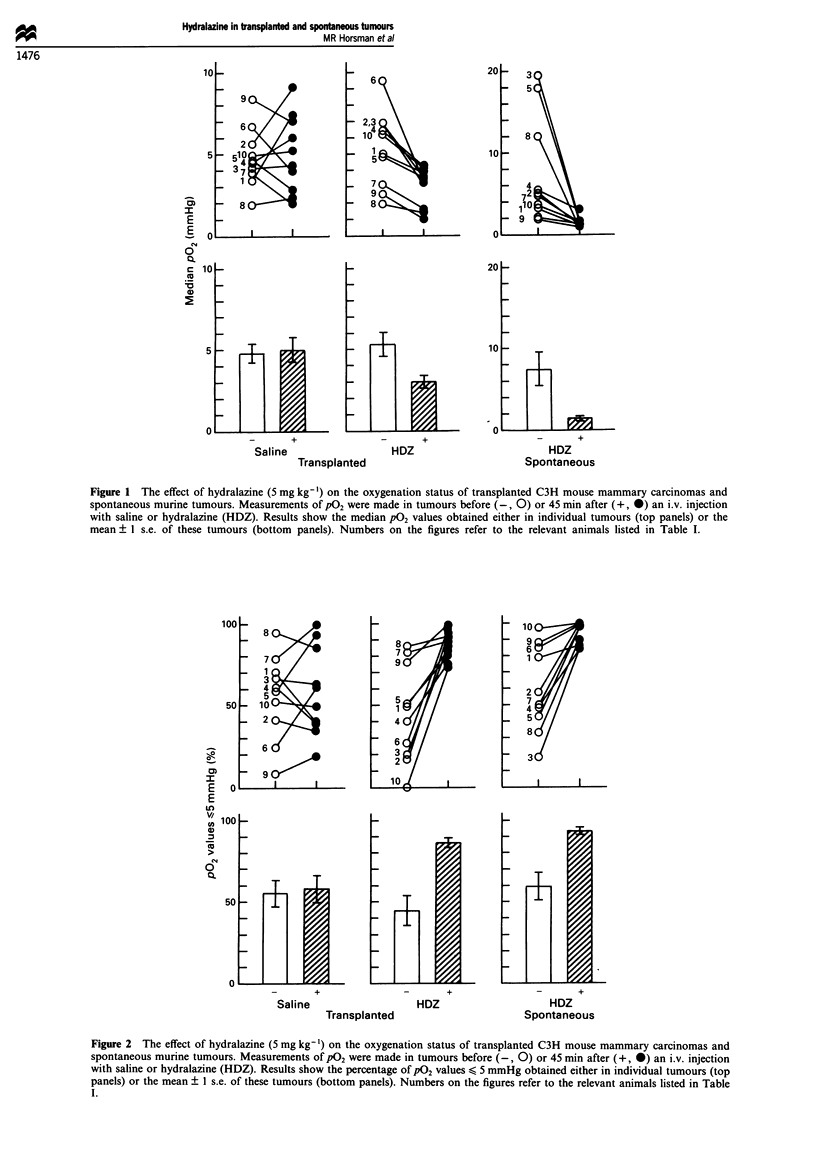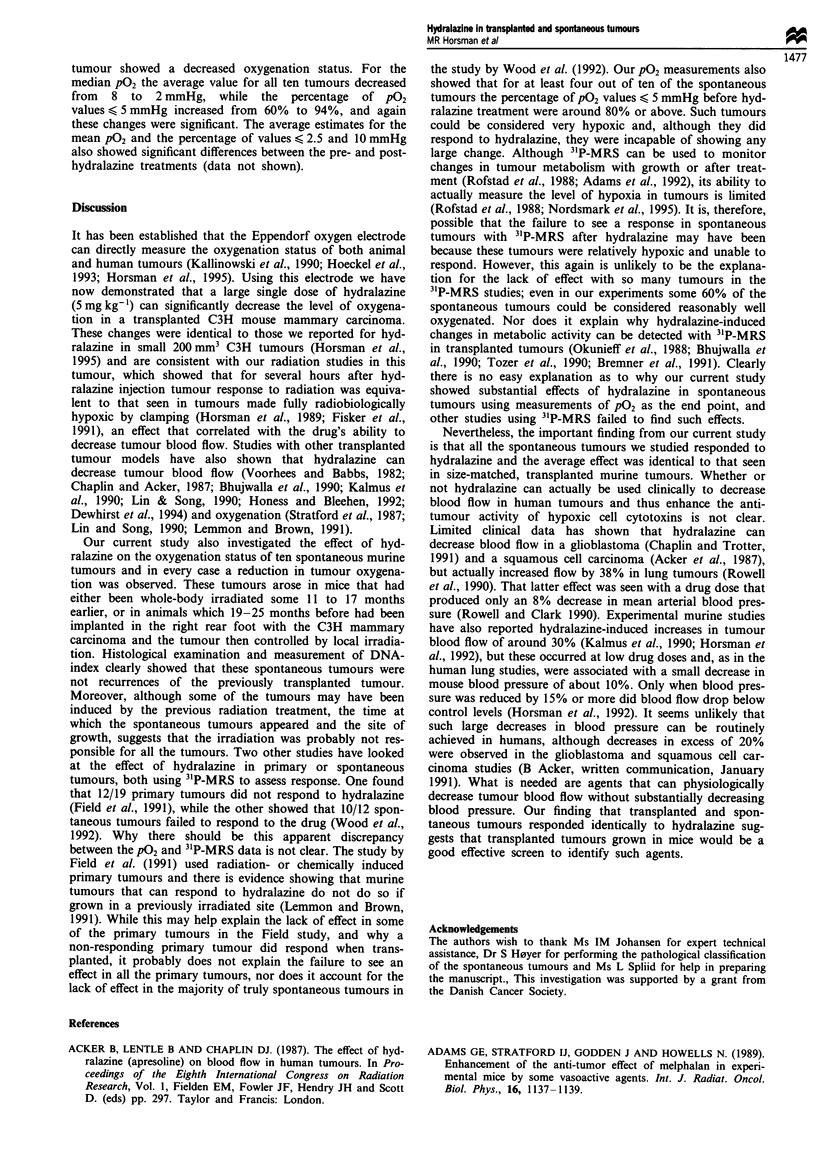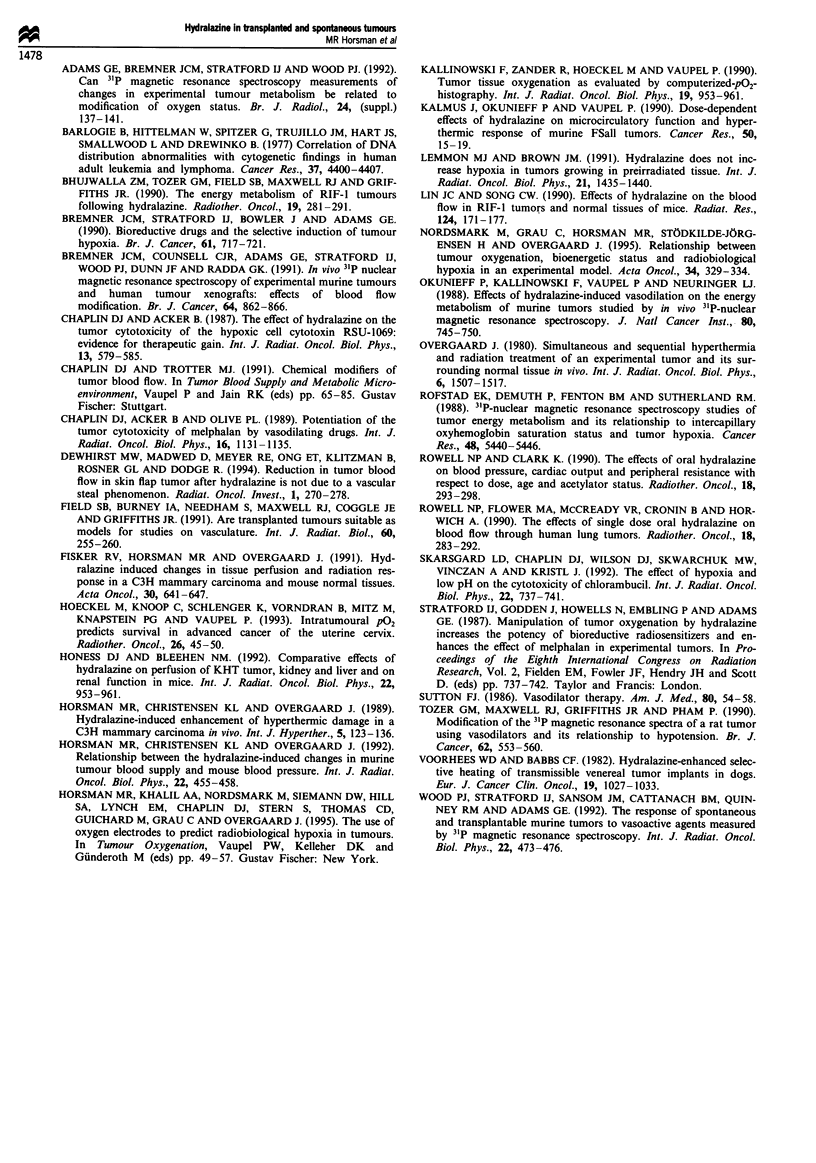# Direct evidence that hydralazine can induce hypoxia in both transplanted and spontaneous murine tumours.

**DOI:** 10.1038/bjc.1995.532

**Published:** 1995-12

**Authors:** M. R. Horsman, M. Nordsmark, M. Høyer, J. Overgaard

**Affiliations:** Danish Cancer Society, Department of Experimental Clinical Oncology, Aarhus, Denmark.

## Abstract

Hydralazine can substantially decrease blood flow and increase hypoxia in transplanted tumours. Previous indirect studies have suggested that hydralazine does not induce such effects in spontaneous tumours. We have now directly investigated the ability of hydralazine to increase hypoxia in both transplanted and spontaneous murine tumours by measuring tumour oxygen partial pressure (pO2) distributions using an Eppendorf oxygen electrode. Spontaneous tumours arose at different sites in CDF1 mice, while transplanted tumours were produced by implanting a C3H mouse mammary carcinoma on the backs of the same mouse strain. Measurements of pO2 were made in anaesthetised mice immediately before and 45 min after an intravenous injection of 5 mg kg-1 hydralazine. In the transplanted tumours hydralazine significantly decreased tumour oxygenation, such that the percentage of pO2 values < or = 5 mmHg increased from 45% to 87%, and median pO2 decreased from 5 to 3 mmHg. Similar significant changes were induced by hydralazine in the spontaneous tumours, the percentage of pO2 values < or = 5 mmHg increasing from 60% to 94% while the median pO2 values decreased from 8 to 2 mmHg. These results clearly show that there is no difference in the response of transplanted and spontaneous mouse tumours to hydralazine.


British Journal of Cancer (1995) 72, 1474-1478
© 1995 Stockton Press All rights reserved 0007-0920/95 $12.00

### Direct evidence that hydralazine can induce hypoxia in both transplanted and spontaneous murine tumours

MR Horsman, M Nordsmark, M Høyer and J Overgaard

*Danish Cancer Society, Department of Experimental Clinical Oncology, Nörrebrogade 44, Bldg. 5, DK-8000 Aarhus C, Denmark.*

**Summary** Hydralazine can substantially decrease blood flow and increase hypoxia in transplanted tumours. Previous indirect studies have suggested that hydralazine does not induce such effects in spontaneous tumours. We have now directly investigated the ability of hydralazine to increase hypoxia in both transplanted and spontaneous murine tumours by measuring tumour oxygen partial pressure ($pO_2$) distributions using an Eppendorf oxygen electrode. Spontaneous tumours arose at different sites in CDF1 mice, while transplanted tumours were produced by implanting a C3H mouse mammary carcinoma on the backs of the same mouse strain. Measurements of $pO_2$ were made in anaesthetised mice immediately before and 45 min after an intravenous injection of 5 mg kg$_{-1}$ hydralazine. In the transplanted tumours hydralazine significantly decreased tumour oxygenation, such that the percentage of $pO_2$ values $\leqslant$ 5 mmHg increased from 45% to 87%, and median $pO_2$ decreased from 5 to 3 mmHg. Similar significant changes were induced by hydralazine in the spontaneous tumours, the percentage of $pO_2$ values $\leqslant$ 5 mmHg increasing from 60% to 94% while the median $pO_2$ values decreased from 8 to 2 mmHg. These results clearly show that there is no difference in the response of transplanted and spontaneous mouse tumours to hydralazine.

**Keywords:** hydralazine; transplanted C3H mouse mammary carcinoma; spontaneous murine tumours; hypoxia; Eppendorf oxygen electrode

It has been well established that the antihypertensive drug hydralazine (Sutton, 1986), when injected into animals at high doses, can substantially decrease blood flow to a variety of transplanted tumours (Voorhees and Babbs, 1982; Chaplin and Acker, 1987; Horsman *et al.*, 1989, 1992; Bhujwalla *et al.*, 1990; Kalmus *et al.*, 1990; Lin and Song, 1990; Fisker *et al.*, 1991; Honess and Bleehen, 1992; Dewhirst *et al.*, 1994). This effect leads to changes in tumour oxygenation (Stratford *et al.*, 1987; Horsman *et al.*, 1989; Lin and Song, 1990; Fisker *et al.*, 1991; Lemmon and Brown, 1991) and energy metabolism (Okunieff *et al.*, 1988; Bhujwalla *et al.*, 1990; Tozer *et al.*, 1990; Bremner *et al.*, 1991), and as a result this drug will enhance the anti-tumour activity of a range of agents. These include hypoxic cell cytotoxins such as bioreductive drugs (Chaplin and Acker, 1987; Bremner *et al.*, 1990) and hyperthermia (Horsman *et al.*, 1989; Kalmus *et al.*, 1990), as well as certain conventional chemotherapeutic agents such as melphalan (Stratford *et al.*, 1987; Adams *et al.*, 1989; Chaplin *et al.*, 1989) and chlorambucil (Skarsgard *et al.*, 1992).

However, recent studies using $^{31}P$ magnetic resonance spectroscopy ($^{31}P$-MRS) reported that primary or spontaneous murine tumours were in general unresponsive to hydralazine (Field *et al.*, 1991; Wood *et al.*, 1992). Moreover, when one of the unresponsive primary tumours was subcutaneously transplanted into the flanks of isogeneic mice it did respond to hydralazine (Field *et al.*, 1991). These findings have not only raised questions about the potential adjuvant use of vascular modifying agents such as hydralazine in clinical therapy, they have also led to criticism about the use of transplanted murine tumours as preclinical screens for testing the activity of such agents.

We have now used an Eppendorf oxygen electrode to measure directly tumour oxygenation, both before and after hydralazine treatment, in a transplanted C3H mouse mammary carcinoma and a series of spontaneous murine tumours. Our results show that hydralazine can in fact significantly decrease oxygenation status in both tumour types.

### Materials and methods

### *Transplanted tumour*

A C3H mouse mammary carcinoma was used. Its derivation and maintenance have been described previously (Overgaard, 1980). Experimental tumours were produced following sterile dissection of large flank tumours. Macroscopically viable tumour tissue was minced with a pair of scissors and 5–10 μl of this material injected subcutaneously on the backs of 10-14-week old male and female C3D2F1/Bom (C3H/Tif female × DBA/2 male) mice. Measurements of tumour oxygenation were carried out when tumours had achieved volumes ranging from about 500 to 5000 mm$^3$ (see Table I), tumour size being determined by the formula $D_1 \times D_2 \times D_3 \times \pi/6$ (where the $D$ values represent three orthogonal diameters). This size range was selected to allow direct comparison with our spontaneous tumours.

### *Spontaneous tumours*

The characteristics of the spontaneous mouse tumours are also listed in Table I. Tumours were used when they had reached about 500–5000 mm$^3$ in size. These volumes were chosen because they were similar to the range of spontaneous tumour volumes used in previous $^{31}P$-MRS studies (Field *et al.*, 1991). Our spontaneous tumours were not deliberately produced for these experiments but arose at different sites in male and female C3D2F1/Bom mice aged between 14 and 28 months that had previously undergone some form of radiation treatment at around 3 months of age in connection with other experiments. Two mice had received whole body irradiation with 6–8 Gy given in three daily fractions. The remaining eight mice had been implanted with a C3H mammary carcinoma in the right rear foot and then locally irradiated with 30–70 Gy when the tumours were $\leqslant$ 200 mm$^3$. Determination of the tumour type was made from microscopic examination of haematoxylin-stained histological sections. DNA index of the tumours was estimated by flow cytometry on ethanol-fixed tumour specimens as described previously (Barlogie *et al.*, 1977).

Correspondence: MR Horsman
Received 30 March 1995; revised 17 July 1995; accepted 8 August 1995

Table 1 Characteristics of the transplanted and spontaneous mouse tumours

| | Transplanted (Controls)[a] | | Transplanted (HDZ treated)[a] | | | | Spontaneous (HDZ treated)[a] | | | | |
|---|---|---|---|---|---|---|---|---|---|---|---|
| Mouse No. | Tumour size ($mm^3$) | Mouse sex | Tumour size ($mm^3$) | Mouse sex | Tumour size ($mm^3$) | Mouse sex | Tumour type | Tumour site | Radiation[b] treatment | Mouse age (months) | DNA index |
| 1 | 509 | F | 509 | F | 507 | F | Malignant lymphoma | Left flank | Foot | 22 | 1.00 |
| 2 | 749 | F | 1056 | M | 545 | M | Sarcoma | Head at right eye | Whole body | 20 | 1.89 |
| 3 | 919 | M | 1144 | M | 848 | F | Necrotic tissue | Right side of neck | Foot | 22 | NA |
| 4 | 968 | F | 1257 | M | 973 | M | Adenocarcinoma | Right breast | Whole body | 14 | 2.37 |
| 5 | 968 | M | 1290 | F | 1131 | F | Adenocarcinoma | Anal–genital region | Foot | 24 | 1.00 |
| 6 | 1021 | F | 1469 | F | 1851 | F | NA | Anal–genital region | Foot | 23 | NA |
| 7 | 2547 | F | 1736 | F | 2118 | F | Adenocarcinoma | Top of right foreleg | Foot | 22 | 1.00 |
| 8 | 2681 | F | 2136 | F | 2706 | F | Adenocarcinoma | Left flank | Foot | 28 | 1.00 |
| 9 | 2893 | F | 2545 | F | 2786 | F | Adenocarcinoma | Left shoulder | Foot | 22 | NA |
| 10 | 5012 | M | 4976 | F | 4838 | F | Anaplastic tumour | Right flank | Foot | 22 | 1.00 |

[a]Mice were intravenously injected with either saline (controls) or hydralazine (HDZ; 5 mg $kg^{-1}$). [b]Mice had previously been irradiated when around 3 months old. Radiation was given either whole body (6–8 Gy in three fractions), or locally to the right rear foot (single dose of 30–70 Gy) in which a C3H mammary carcinoma had been implanted. NA, not available.

### *Drug preparation*

Hydralazine (1-hydrazinophthalazine) was supplied by Ciba-Geigy, Copenhagen, Denmark. A fresh solution was prepared in saline (0.9% sodium chloride) before each series of measurements. It was then injected intravenously (i.v.) at a constant injection volume of 0.02 ml $g^{-1}$ mouse body weight.

### *$pO_2$ measurements*

Measurements of tumour $pO_2$ were made using a computerised fine-needle polarographic oxygen electrode probe (Eppendorf, Hamburg, Germany), the details of which have been described previously (Kallinowski *et al.*, 1990). The location of some of the spontaneous tumours required the use of an anaesthetic, thus for consistency $pO_2$ measurements in all the spontaneous and transplanted tumours were performed under anaesthesia. To achieve this mice were given a single injection with a mixture of hypnorm (fluanisonum 10 mg $ml^{-1}$ + fentanyl 0.2 mg $ml^{-1}$), diazepam (5 mg $ml^{-1}$) and water in the ratio of 1:1:4. This mixture was injected intraperitoneally at 0.005 ml $g^{-1}$ mouse body weight. The oxygen electrode was then inserted up to a depth of 1 mm into the tumour. It was subsequently moved automatically through the tissue in 0.7 mm increments, followed each time by a 0.3 mm backward step before measurement. Between two and six repeated parallel insertions were performed in each tumour. Mice were then injected with saline or hydralazine and 45 min later the $pO_2$ measurements were repeated, the animals remaining anaesthetised for this entire period. The average number of $pO_2$ values obtained per set of measurements was 120 (range 37–220). Body temperature was monitored using a rectally inserted thermocouple before hydralazine injection and at various times after, and maintained at the prehydralazine level by warming the mice from above with a standard desk-top lamp.

### *Data analysis*

The relative frequency of the $pO_2$ measurements was automatically calculated and displayed as a histogram, but from the original raw data a number of parameters can be selected to reflect tumour oxygenation. In this study we decided to show the results from only two of these end points, namely, median $pO_2$ and percentage of $pO_2$ values $\leqslant$ 5 mmHg, because the former is indicative of the overall oxygenation status, while the latter is likely to include all the radiobiologically hypoxic cells in the tumour. Statistical analysis of the data was performed using the Student *t*-test after testing for variance homogeneity by an *F*-test, with $P = 0.05$ selected as the level of significance.

### Results

Histological characterisation of the spontaneous tumours showed that half of them were adenocarcinomas (Table I). The remainder being a malignant lymphoma, a sarcoma, an anaplastic tumour and one tumour (mouse number 3) which histologically was actually found to consist of highly vascularised, yet necrotic tissue, with no malignant cells being seen. Seven of the tumours also had their DNA-index determined and of these, five were diploid (DNA index of 1.00), while the remaining two were either hypotetraploid (mouse number 2) or hypertetraploid (mouse number 4). The transplanted C3H mouse mammary carcinoma was found to be tetraploid with a DNA index of 2.01.

Figures 1 and 2 show the median $pO_2$ and percentage of $pO_2$ values $\leqslant$ 5 mmHg, before and after drug injection, for all the transplanted and spontaneous tumours used in this study. To check on the validity of making repeated $pO_2$ measurements in tumours, measurements were made in transplanted tumours before and after injection with saline. The pretreatment median $pO_2$ values ranged from 2 to 8 mmHg (mean = 5 mmHg) and the percentage of $pO_2$ values $\leqslant$ 5 mmHg ranged from 8% to 94% (mean = 55%). Following injection with saline the $pO_2$ measurements were repeated and although there was some variability between the pre- and post-saline measurements, there were no consistent changes. Moreover, the post-injection results covered a similar range (median values went from 2 to 9 mmHg and the percentage $\leqslant$ 5 mmHg from 19% to 99%) as the presaline measurements, and the average estimates of the median $pO_2$ values and percentage of $pO_2$ values $\leqslant$ 5 mmHg were calculated to be 5 mmHg and 58% respectively, which were not significantly different from the average presaline values.

The pretreatment $pO_2$ values measured in the transplanted tumours used for the hydralazine study were almost identical to those seen in the saline-treated transplanted tumours. However, when mice were injected with hydralazine every single transplanted tumour became less well oxygenated. Indeed, for all ten tumours the average of the median $pO_2$ values was decreased from 5 to 3 mmHg, while the percentage of $pO_2$ values $\leqslant$ 5 mmHg increased from 45% to 87%, and these changes in both parameters were significant. Similar significant changes were also seen for mean $pO_2$ and the percentage of values $\leqslant$ 10 mmHg, but not for the percentage $\leqslant$ 2.5 mmHg (data not shown).

For the spontaneous tumours the prehydralazine treatment $pO_2$ values did appear to show a wider range, at least in terms of median $pO_2$, than the pretreatment values measured in transplanted tumours, however, the average results for all ten spontaneous tumours were not significantly different from the average pretreatment $pO_2$ estimates of the transplanted tumours. After hydralazine treatment, every spontaneous

**Figure 1** The effect of hydralazine (5 mg $kg^{-1}$) on the oxygenation status of transplanted C3H mouse mammary carcinomas and spontaneous murine tumours. Measurements of $pO_2$ were made in tumours before (–, ○) or 45 min after (+, ●) an i.v. injection with saline or hydralazine (HDZ). Results show the median $pO_2$ values obtained either in individual tumours (top panels) or the mean ± 1 s.e. of these tumours (bottom panels). Numbers on the figures refer to the relevant animals listed in Table I.

**Figure 2** The effect of hydralazine (5 mg $kg^{-1}$) on the oxygenation status of transplanted C3H mouse mammary carcinomas and spontaneous murine tumours. Measurements of $pO_2$ were made in tumours before (–, ○) or 45 min after (+, ●) an i.v. injection with saline or hydralazine (HDZ). Results show the percentage of $pO_2$ values ≤ 5 mmHg obtained either in individual tumours (top panels) or the mean ± 1 s.e. of these tumours (bottom panels). Numbers on the figures refer to the relevant animals listed in Table I.

tumour showed a decreased oxygenation status. For the median $pO_2$ the average value for all ten tumours decreased from 8 to 2 mmHg, while the percentage of $pO_2$ values $\leqslant$ 5 mmHg increased from 60% to 94%, and again these changes were significant. The average estimates for the mean $pO_2$ and the percentage of values $\leqslant$ 2.5 and 10 mmHg also showed significant differences between the pre- and post-hydralazine treatments (data not shown).

### Discussion

It has been established that the Eppendorf oxygen electrode can directly measure the oxygenation status of both animal and human tumours (Kallinowski *et al.*, 1990; Hoeckel *et al.*, 1993; Horsman *et al.*, 1995). Using this electrode we have now demonstrated that a large single dose of hydralazine (5 mg $kg^{-1}$) can significantly decrease the level of oxygenation in a transplanted C3H mouse mammary carcinoma. These changes were identical to those we reported for hydralazine in small 200 $mm^3$ C3H tumours (Horsman *et al.*, 1995) and are consistent with our radiation studies in this tumour, which showed that for several hours after hydralazine injection tumour response to radiation was equivalent to that seen in tumours made fully radiobiologically hypoxic by clamping (Horsman *et al.*, 1989; Fisker *et al.*, 1991), an effect that correlated with the drug's ability to decrease tumour blood flow. Studies with other transplanted tumour models have also shown that hydralazine can decrease tumour blood flow (Voorhees and Babbs, 1982; Chaplin and Acker, 1987; Bhujwalla *et al.*, 1990; Kalmus *et al.*, 1990; Lin & Song, 1990; Honess and Bleehen, 1992; Dewhirst *et al.*, 1994) and oxygenation (Stratford *et al.*, 1987; Lin and Song, 1990; Lemmon and Brown, 1991).

Our current study also investigated the effect of hydralazine on the oxygenation status of ten spontaneous murine tumours and in every case a reduction in tumour oxygenation was observed. These tumours arose in mice that had either been whole-body irradiated some 11 to 17 months earlier, or in animals which 19–25 months before had been implanted in the right rear foot with the C3H mammary carcinoma and the tumour then controlled by local irradiation. Histological examination and measurement of DNA-index clearly showed that these spontaneous tumours were not recurrences of the previously transplanted tumour. Moreover, although some of the tumours may have been induced by the previous radiation treatment, the time at which the spontaneous tumours appeared and the site of growth, suggests that the irradiation was probably not responsible for all the tumours. Two other studies have looked at the effect of hydralazine in primary or spontaneous tumours, both using $^{31}$P-MRS to assess response. One found that 12/19 primary tumours did not respond to hydralazine (Field *et al.*, 1991), while the other showed that 10/12 spontaneous tumours failed to respond to the drug (Wood *et al.*, 1992). Why there should be this apparent discrepancy between the $pO_2$ and $^{31}$P-MRS data is not clear. The study by Field *et al.* (1991) used radiation- or chemically induced primary tumours and there is evidence showing that murine tumours that can respond to hydralazine do not do so if grown in a previously irradiated site (Lemmon and Brown, 1991). While this may help explain the lack of effect in some of the primary tumours in the Field study, and why a non-responding primary tumour did respond when transplanted, it probably does not explain the failure to see an effect in all the primary tumours, nor does it account for the lack of effect in the majority of truly spontaneous tumours in the study by Wood *et al.* (1992). Our $pO_2$ measurements also showed that for at least four out of ten of the spontaneous tumours the percentage of $pO_2$ values $\leqslant$ 5 mmHg before hydralazine treatment were around 80% or above. Such tumours could be considered very hypoxic and, although they did respond to hydralazine, they were incapable of showing any large change. Although $^{31}$P-MRS can be used to monitor changes in tumour metabolism with growth or after treatment (Rofstad *et al.*, 1988; Adams *et al.*, 1992), its ability to actually measure the level of hypoxia in tumours is limited (Rofstad *et al.*, 1988; Nordsmark *et al.*, 1995). It is, therefore, possible that the failure to see a response in spontaneous tumours with $^{31}$P-MRS after hydralazine may have been because these tumours were relatively hypoxic and unable to respond. However, this again is unlikely to be the explanation for the lack of effect with so many tumours in the $^{31}$P-MRS studies; even in our experiments some 60% of the spontaneous tumours could be considered reasonably well oxygenated. Nor does it explain why hydralazine-induced changes in metabolic activity can be detected with $^{31}$P-MRS in transplanted tumours (Okunieff *et al.*, 1988; Bhujwalla *et al.*, 1990; Tozer *et al.*, 1990; Bremner *et al.*, 1991). Clearly there is no easy explanation as to why our current study showed substantial effects of hydralazine in spontaneous tumours using measurements of $pO_2$ as the end point, and other studies using $^{31}$P-MRS failed to find such effects.

Nevertheless, the important finding from our current study is that all the spontaneous tumours we studied responded to hydralazine and the average effect was identical to that seen in size-matched, transplanted murine tumours. Whether or not hydralazine can actually be used clinically to decrease blood flow in human tumours and thus enhance the anti-tumour activity of hypoxic cell cytotoxins is not clear. Limited clinical data has shown that hydralazine can decrease blood flow in a glioblastoma (Chaplin and Trotter, 1991) and a squamous cell carcinoma (Acker *et al.*, 1987), but actually increased flow by 38% in lung tumours (Rowell *et al.*, 1990). That latter effect was seen with a drug dose that produced only an 8% decrease in mean arterial blood pressure (Rowell and Clark 1990). Experimental murine studies have also reported hydralazine-induced increases in tumour blood flow of around 30% (Kalmus *et al.*, 1990; Horsman *et al.*, 1992), but these occurred at low drug doses and, as in the human lung studies, were associated with a small decrease in mouse blood pressure of about 10%. Only when blood pressure was reduced by 15% or more did blood flow drop below control levels (Horsman *et al.*, 1992). It seems unlikely that such large decreases in blood pressure can be routinely achieved in humans, although decreases in excess of 20% were observed in the glioblastoma and squamous cell carcinoma studies (B Acker, written communication, January 1991). What is needed are agents that can physiologically decrease tumour blood flow without substantially decreasing blood pressure. Our finding that transplanted and spontaneous tumours responded identically to hydralazine suggests that transplanted tumours grown in mice would be a good effective screen to identify such agents.

### Acknowledgements

The authors wish to thank Ms IM Johansen for expert technical assistance, Dr S Høyer for performing the pathological classification of the spontaneous tumours and Ms L Spliid for help in preparing the manuscript., This investigation was supported by a grant from the Danish Cancer Society.

### References

ACKER B, LENTLE B AND CHAPLIN DJ. (1987). The effect of hydralazine (apresoline) on blood flow in human tumours. In *Proceedings of the Eighth International Congress on Radiation Research*, Vol. 1, Fielden EM, Fowler JF, Hendry JH and Scott D. (eds) pp. 297. Taylor and Francis: London.

ADAMS GE, STRATFORD IJ, GODDEN J AND HOWELLS N. (1989). Enhancement of the anti-tumor effect of melphalan in experimental mice by some vasoactive agents. *Int. J. Radiat. Oncol. Biol. Phys.*, **16**, 1137–1139.

ADAMS GE, BREMNER JCM, STRATFORD IJ AND WOOD PJ. (1992). Can $^{31}$P magnetic resonance spectroscopy measurements of changes in experimental tumour metabolism be related to modification of oxygen status. *Br. J. Radiol.*, **24**, (suppl.) 137–141.
BARLOGIE B, HITTELMAN W, SPITZER G, TRUJILLO JM, HART JS, SMALLWOOD L AND DREWINKO B. (1977) Correlation of DNA distribution abnormalities with cytogenetic findings in human adult leukemia and lymphoma. *Cancer Res.*, **37**, 4400–4407.
BHUJWALLA ZM, TOZER GM, FIELD SB, MAXWELL RJ AND GRIFFITHS JR. (1990). The energy metabolism of RIF-1 tumours following hydralazine. *Radiother. Oncol.*, **19**, 281–291.
BREMNER JCM, STRATFORD IJ, BOWLER J AND ADAMS GE. (1990). Bioreductive drugs and the selective induction of tumour hypoxia. *Br. J. Cancer*, **61**, 717–721.
BREMNER JCM, COUNSELL CJR, ADAMS GE, STRATFORD IJ, WOOD PJ, DUNN JF AND RADDA GK. (1991). *In vivo* $^{31}$P nuclear magnetic resonance spectroscopy of experimental murine tumours and human tumour xenografts: effects of blood flow modification. *Br. J. Cancer*, **64**, 862–866.
CHAPLIN DJ AND ACKER B. (1987). The effect of hydralazine on the tumor cytotoxicity of the hypoxic cell cytotoxin RSU-1069: evidence for therapeutic gain. *Int. J. Radiat. Oncol. Biol. Phys.*, **13**, 579–585.
CHAPLIN DJ AND TROTTER MJ. (1991). Chemical modifiers of tumor blood flow. In *Tumor Blood Supply and Metabolic Microenvironment*, Vaupel P and Jain RK (eds) pp. 65–85. Gustav Fischer: Stuttgart.
CHAPLIN DJ, ACKER B AND OLIVE PL. (1989). Potentiation of the tumor cytotoxicity of melphalan by vasodilating drugs. *Int. J. Radiat. Oncol. Biol. Phys.*, **16**, 1131–1135.
DEWHIRST MW, MADWED D, MEYER RE, ONG ET, KLITZMAN B, ROSNER GL AND DODGE R. (1994). Reduction in tumor blood flow in skin flap tumor after hydralazine is not due to a vascular steal phenomenon. *Radiat. Oncol. Invest.*, **1**, 270–278.
FIELD SB, BURNEY IA, NEEDHAM S, MAXWELL RJ, COGGLE JE AND GRIFFITHS JR. (1991). Are transplanted tumours suitable as models for studies on vasculature. *Int. J. Radiat. Biol.*, **60**, 255–260.
FISKER RV, HORSMAN MR AND OVERGAARD J. (1991). Hydralazine induced changes in tissue perfusion and radiation response in a C3H mammary carcinoma and mouse normal tissues. *Acta Oncol.*, **30**, 641–647.
HOECKEL M, KNOOP C, SCHLENGER K, VORNDRAN B, MITZ M, KNAPSTEIN PG AND VAUPEL P. (1993). Intratumoural $pO_2$ predicts survival in advanced cancer of the uterine cervix. *Radiother. Oncol.*, **26**, 45–50.
HONESS DJ AND BLEEHEN NM. (1992). Comparative effects of hydralazine on perfusion of KHT tumor, kidney and liver and on renal function in mice. *Int. J. Radiat. Oncol. Biol. Phys.*, **22**, 953–961.
HORSMAN MR, CHRISTENSEN KL AND OVERGAARD J. (1989). Hydralazine-induced enhancement of hyperthermic damage in a C3H mammary carcinoma *in vivo*. *Int. J. Hyperther.*, **5**, 123–136.
HORSMAN MR, CHRISTENSEN KL AND OVERGAARD J. (1992). Relationship between the hydralazine-induced changes in murine tumour blood supply and mouse blood pressure. *Int. J. Radiat. Oncol. Biol. Phys.*, **22**, 455–458.
HORSMAN MR, KHALIL AA, NORDSMARK M, SIEMANN DW, HILL SA, LYNCH EM, CHAPLIN DJ, STERN S, THOMAS CD, GUICHARD M, GRAU C AND OVERGAARD J. (1995). The use of oxygen electrodes to predict radiobiological hypoxia in tumours. In *Tumour Oxygenation*, Vaupel PW, Kelleher DK and Günderoth M (eds) pp. 49–57. Gustav Fischer: New York.
KALLINOWSKI F, ZANDER R, HOECKEL M AND VAUPEL P. (1990). Tumor tissue oxygenation as evaluated by computerized-$pO_2$-histography. *Int. J. Radiat. Oncol. Biol. Phys.*, **19**, 953–961.
KALMUS J, OKUNIEFF P AND VAUPEL P. (1990). Dose-dependent effects of hydralazine on microcirculatory function and hyperthermic response of murine FSall tumors. *Cancer Res.*, **50**, 15–19.
LEMMON MJ AND BROWN JM. (1991). Hydralazine does not increase hypoxia in tumors growing in preirradiated tissue. *Int. J. Radiat. Oncol. Biol. Phys.*, **21**, 1435–1440.
LIN JC AND SONG CW. (1990). Effects of hydralazine on the blood flow in RIF-1 tumors and normal tissues of mice. *Radiat. Res.*, **124**, 171–177.
NORDSMARK M, GRAU C, HORSMAN MR, STÖDKILDE-JÖRGENSEN H AND OVERGAARD J. (1995). Relationship between tumour oxygenation, bioenergetic status and radiobiological hypoxia in an experimental model. *Acta Oncol.*, **34**, 329–334.
OKUNIEFF P, KALLINOWSKI F, VAUPEL P AND NEURINGER LJ. (1988). Effects of hydralazine-induced vasodilation on the energy metabolism of murine tumors studied by *in vivo* $^{31}$P-nuclear magnetic resonance spectroscopy. *J. Natl Cancer Inst.*, **80**, 745–750.
OVERGAARD J. (1980). Simultaneous and sequential hyperthermia and radiation treatment of an experimental tumor and its surrounding normal tissue *in vivo*. *Int. J. Radiat. Oncol. Biol. Phys.*, **6**, 1507–1517.
ROFSTAD EK, DEMUTH P, FENTON BM AND SUTHERLAND RM. (1988). $^{31}$P-nuclear magnetic resonance spectroscopy studies of tumor energy metabolism and its relationship to intercapillary oxyhemoglobin saturation status and tumor hypoxia. *Cancer Res.*, **48**, 5440–5446.
ROWELL NP AND CLARK K. (1990). The effects of oral hydralazine on blood pressure, cardiac output and peripheral resistance with respect to dose, age and acetylator status. *Radiother. Oncol.*, **18**, 293–298.
ROWELL NP, FLOWER MA, MCCREADY VR, CRONIN B AND HORWICH A. (1990). The effects of single dose oral hydralazine on blood flow through human lung tumors. *Radiother. Oncol.*, **18**, 283–292.
SKARSGARD LD, CHAPLIN DJ, WILSON DJ, SKWARCHUK MW, VINCZAN A AND KRISTL J. (1992). The effect of hypoxia and low pH on the cytotoxicity of chlorambucil. *Int. J. Radiat. Oncol. Biol. Phys.*, **22**, 737–741.
STRATFORD IJ, GODDEN J, HOWELLS N, EMBLING P AND ADAMS GE. (1987). Manipulation of tumor oxygenation by hydralazine increases the potency of bioreductive radiosensitizers and enhances the effect of melphalan in experimental tumors. In *Proceedings of the Eighth International Congress on Radiation Research*, Vol. 2, Fielden EM, Fowler JF, Hendry JH and Scott D. (eds) pp. 737–742. Taylor and Francis: London.
SUTTON FJ. (1986). Vasodilator therapy. *Am. J. Med.*, **80**, 54–58.
TOZER GM, MAXWELL RJ, GRIFFITHS JR AND PHAM P. (1990). Modification of the $^{31}$P magnetic resonance spectra of a rat tumor using vasodilators and its relationship to hypotension. *Br. J. Cancer*, **62**, 553–560.
VOORHEES WD AND BABBS CF. (1982). Hydralazine-enhanced selective heating of transmissible venereal tumor implants in dogs. *Eur. J. Cancer Clin. Oncol.*, **19**, 1027–1033.
WOOD PJ, STRATFORD IJ, SANSOM JM, CATTANACH BM, QUINNEY RM AND ADAMS GE. (1992). The response of spontaneous and transplantable murine tumors to vasoactive agents measured by $^{31}$P magnetic resonance spectroscopy. *Int. J. Radiat. Oncol. Biol. Phys.*, **22**, 473–476.